# Gasdermin D promotes hyperinflammation and immunopathology during severe influenza A virus infection

**DOI:** 10.1038/s41419-023-06258-1

**Published:** 2023-11-09

**Authors:** Sarah Rosli, Christopher M. Harpur, Maggie Lam, Alison C. West, Christopher Hodges, Ashley Mansell, Kate E. Lawlor, Michelle D. Tate

**Affiliations:** 1 Centre for Innate Immunity and Infectious Diseases, Hudson Institute of Medical Research, Clayton, Vic Australia; 2 Department of Molecular and Translational Sciences, Monash University, Clayton, Vic Australia; 3 Adiso Therapeutics, Concord, MA USA

**Keywords:** Infection, Inflammasome

## Abstract

Excessive inflammation and tissue damage during severe influenza A virus (IAV) infection can lead to the development of fatal pulmonary disease. Pyroptosis is a lytic and pro-inflammatory form of cell death executed by the pore-forming protein gasdermin D (GSDMD). In this study, we investigated a potential role for GSDMD in promoting the development of severe IAV disease. IAV infection resulted in cleavage of GSDMD in vivo and in vitro in lung epithelial cells. Mice genetically deficient in GSDMD (*Gsdmd*^−/−^) developed less severe IAV disease than wildtype mice and displayed improved survival outcomes. GSDMD deficiency significantly reduced neutrophil infiltration into the airways as well as the levels of pro-inflammatory cytokines TNF, IL-6, MCP-1, and IL-1α and neutrophil-attracting chemokines CXCL1 and CXCL2. In contrast, IL-1β and IL-18 responses were not largely impacted by GSDMD deficiency. In addition, *Gsdmd*^−/−^ mice displayed significantly improved influenza disease resistance with reduced viral burden and less severe pulmonary pathology, including decreased epithelial damage and cell death. These findings indicate a major role for GSDMD in promoting damaging inflammation and the development of severe IAV disease.

## Introduction

Pandemic and seasonal influenza A viruses (IAV) pose a strong ongoing threat to human health globally [1]. Severe IAV infections are associated with excessive inflammation, cell death, and damage to the epithelium, which contribute to the development of untreatable and fatal lung disease [2, 3]. While the molecular mechanisms involved in the induction of inflammation during IAV infection have been extensively studied, the pathways involved in IAV-induced cell death and their impact on inflammation and immunopathology are less well described [2, 3]. A greater understanding of the role of cell death in host defense and disease pathogenesis may facilitate the design of new and effective host-directed therapies for severe IAV infections.

NOD-, LRR- and pyrin domain-containing protein 3 (NLRP3) inflammasomes are cytosolic innate immune complexes that assemble during IAV infection in response to viral and endogenous cellular danger signals [2, 3]. These include pathogen-associated molecular patterns (PAMPs, e.g., viral RNA, IAV PB1-F2, and M2 proteins), as well as danger-associated molecular patterns (DAMPs, e.g., extracellular ATP and IL-1α) [4–7]. Activation of the NLRP3 inflammasome complex, comprising NLRP3, ASC, and pro-caspase-1, induces proximity-mediated dimerization and activation of caspase-1 to process the inactive cytokines, pro-IL-1β and pro-IL-18, into their bioactive, inflammatory forms, IL-1β and IL-18 [2]. Critically, we have previously shown the NLRP3 inflammasome and IL-1β can promote hyperinflammation in vivo and the development of severe IAV disease [7–9].

NLRP3 inflammasome activation is known to typically result in pyroptosis, a lytic and pro-inflammatory form of cell death [3, 10]. Specifically, NLRP3 inflammasome activation induces caspase-1 cleavage of gasdermin D (GSDMD) between the autoinhibitory C-terminal and active N-terminal (NT) domains (at Asp 276 in mice and Asp 275 in humans) [11]. Active NT GSDMD p30 subunits insert into the lipid membrane and oligomerize to form a transmembrane pore [10, 12]. The GSDMD pore has been shown to facilitate the release of active pro-inflammatory cytokines IL-1β and IL-18 from macrophages and dendritic cells [13, 14]. Ultimately, GSDMD pore formation leads to osmotic cell lysis and the release of inflammatory cellular contents, including DAMPs and PAMPs. Interestingly, Corry et al. detected cleaved GSDMD in the lungs of macaques infected with lethal avian H5N1 IAV [15], suggesting IAV infection induces GSDMD activation. While GSDMD has been shown to play a key role in numerous inflammatory diseases [10], its role in modulating inflammation and the severity of IAV disease in vivo is not currently well understood.

In this study, we investigated a potential role for GSDMD in modulating the severity of IAV infection. Here we show that IAV infection induced GSDMD cleavage in vivo in murine lung epithelial cells and in vitro in human bronchial epithelial cells. Additionally, genetic deficiency of GSDMD limited the severity of IAV disease and improved survival and recovery from infection, which correlated with reduced lung viral burden, as well as diminished neutrophil infiltration and production of pro-inflammatory cytokines TNF, IL-6, MCP-1, and IL-1α in the airways. Finally, mice lacking GSDMD displayed reduced pulmonary pathology, including epithelial damage and cell death. Collectively, our data provides evidence that GSDMD plays a major role in augmenting cell death and hyperinflammation at the site of infection, thereby promoting severe IAV disease.

## Materials and methods

### Influenza virus

IAV strains used were HKx31 (H3N2), which is a high-yielding reassortant of A/PR/8 that carries the surface glycoproteins of A/Aichi/2/1968 (H3N2). In some in vitro experiments, human influenza strains A/Brazil/11/78 (BR; H1N1), A/Solomon Island/3/2006 (SI; H1N1), A/Tasmania/2004/2009 (Tas; pandemic H1N1), and A/Perth/16/2009 (Perth; H3N2) were used. IAV were grown in 10-day embryonated chicken eggs by standard procedures and titrated on Madin-Darby Canine Kidney (MDCK) cells. All viral stocks were stored at −80 °C.

### In vitro IAV infection

Human normal bronchial epithelial HBEC3-KT cells (immortalized and non-malignant) were purchased from ATCC (Virginia, USA) and cultured under submerged conditions on collagen-coated flasks (Thermo Fisher Scientific, Waltham, Massachusetts, USA) in supplemented bronchial epithelial growth medium (BEGM; Lonza, Basel, Switzerland). HBEC3-KT cells were plated into collagen-coated 24-well plates in BEGM medium without hydrocortisone. The following day, cell monolayers were infected with IAV in BEGM media for 1 hr at a multiplicity of infection (MOI) of 3. Cell monolayers were then washed and incubated in media for 24 h. Cell supernatants and cell lysates were collected and stored at −80 °C for protein analysis via immunoblot. Cell supernatants were assayed for levels of lactate dehydrogenase (LDH) using a CytoTox 96 Non-radioactive Cytotoxicity Assay (Promega, Madison, USA), according to the manufacturer’s instructions. Levels of IL-1β and IL-18 in cell supernatants were determined by ELISA (R&D Systems, USA).

### Influenza virus infection of mice

Wildtype and *Gsdmd*^−/−^ C57BL/6N mice (male and female, 6–8 weeks old) were maintained in the Specific Pathogen Free Physical Containment Level 2 (PC2) Animal Research Facility at the Monash Medical Centre (Clayton, Victoria, Australia). *Gsdmd*^−/−^ mice were kindly provided by VM Dixit (Genentech, South San Francisco, California, USA) [16]. All experimental procedures were approved by the Hudson Animal Ethics Committee, and experimental procedures were carried out in accordance with approved guidelines.

For virus infection studies, cages of mice were randomly allocated to groups. Mice were lightly anesthetized with isoflurane, and intranasally inoculated with 10^4^ plaque-forming units (PFU) of HKx31 (H3N2) IAV in 50 µL PBS, which induces severe disease [17]. Mice were weighed daily and assessed for clinical signs of disease on a scale of 0 to 3 (0 = no visible signs; 1 = slight ruffling of fur; 2 = ruffled fur, reduced mobility; 3 = ruffled fur, reduced mobility, rapid breathing). Animals that lost 20% of their original body weight and/or displayed severe clinical signs of disease (a score of 3) were euthanized.

At the indicated time points, mice were sacrificed via intraperitoneal injection of sodium pentobarbital. Bronchoalveolar lavage (BAL) was immediately performed by flushing the lungs three times with 1 mL of PBS. Lung tissues were then removed and frozen immediately in liquid nitrogen. Titres of infectious virus in lung homogenates were determined by standard plaque assay on MDCK cells. In some experiments, whole lung tissues (i.e., no BAL was performed) were excised from mice and immediately frozen in liquid nitrogen for protein analysis.

### Examination of protein expression by immunoblot

Whole lung tissues were homogenized in lysis buffer consisting of 250 mM Tris-HCl (pH 6.8), 10% (w/v) SDS, 20% (v/v) glycerol, supplemented with cOmplete™ Protease Inhibitor (Roche, Basel Switzerland). Protein estimation was used for whole lung samples to normalize protein loading (Bio-Rad DC Protein Assay, Bio-Rad, Hercules, California, USA). Proteins from mouse BAL cells and human HBEC3-KT cell lysates were isolated with RIPA buffer consisting of 50 mM Tris-HCl (pH 8), 150 mM NaCl, 1 mM EDTA, 1% (v/v) Igepal, 0.5% (w/v) Sodium Deoxycholate, 0.1% (w/v) SDS, 10 mM Sodium Fluoride, 1 mM Sodium Orthovanadate, and 1 mM phenylmethylsulfonyl fluoride (Sigma Aldrich, St. Louis, Missouri, USA). Protein lysates from HBEC3-KT cell supernatants and mouse BAL fluids were concentrated using Strataclean resin (Agilent Technologies, Santa Clara, California, USA).

Protein lysates were resolved by 4–12% SDS-PAGE (Life Technologies, Carlsbad, California, USA) and transferred onto PVDF (Merck Millipore, Burlington, Massachusetts, USA). Membranes were blocked with 5% bovine serum albumin (BSA; Sigma Aldrich) in Tris-buffered saline with 0.05% Tween 20 (TBST; Sigma Aldrich) followed by incubation with the desired primary antibodies overnight: anti-mouse Gsdmd (EPR19828; Abcam, Cambridge, UK), anti-human GSDMD (G7422, Sigma Aldrich), anti-human caspase 1 (D7F10; Cell Signaling Technology, Danvers, Massachusetts, USA), anti-mouse IL-1β (clone 30311; R&D Systems, Minneapolis, USA), or anti-mouse alpha tubulin (clone YL1/2; Abcam). Membranes were subsequently probed with the appropriate secondary antibodies and visualized on the Bio-Rad ChemiDoc MP Imaging System (Bio-Rad) via chemiluminescence (human and mouse GSDMD, caspase 1) or fluorescence (IL-1β and alpha tubulin). All original western blots are included in the Supplemental Material.

### Quantification of cytokines and chemokines in mouse BAL fluid

BAL fluid was collected following centrifugation and stored at −80 °C. Levels of IL-6, MCP-1, IFNγ, IL-10, IL-12p70, and TNF proteins were determined by cytokine bead array (CBA) using the mouse inflammation kit (BD Biosciences, San Jose California, USA). Mouse IL-1β, IL-18, IL-1α, KC, and MIP-2 were quantified by ELISA (R&D Systems). Levels of IFNβ and IFNα were measured by ELISA as previously described [18].

### Flow cytometry on mouse BAL cells

Cells in the BAL fluid were isolated by centrifugation and treated with red blood cell lysis buffer (Sigma Aldrich) for 5 min. The reaction was quenched by washing the cells in FACS buffer (PBS containing 2% (v/v) FBS and 2 mM EDTA). BAL cells were then incubated with fluorescently labeled antibodies at 4 °C for 20 min in the presence of Fc receptor blocking monoclonal antibodies against CD16/CD32 (clone 93, Thermo Fisher Scientific) to limit non-specific antibody binding. Specifically, monoclonal antibodies to Siglec-F (clone E50-2440, BD Biosciences), NK1.1 (clone PK136, BioLegend, San Diego, California, USA), CD3ε (clone 145-2C11, BioLegend), CD11c (clone HL3, BD Biosciences), CD64 (clone X54-5/7.1, BioLegend), Ly6C (clone AL-21, BD Biosciences), Ly6G (clone 1A8, BD Biosciences), and I-A^b^ (clone AF6-120.1, BD Biosciences) and the Zombie Aqua viability dye (BioLegend). Total live cells (Zombie Aqua viability dye^-^), neutrophils (Ly6G^+^ Ly6C^int^), NK cells (NK1.1^+^ CD3^−^), T cells (NK1.1^−^ CD3^+^), IM (Ly6G^−^ Ly6C^+^), AM (CD11c^+^ Siglec-F^+^) and DCs (CD11c^+^ I-A^b+^) were quantified by flow cytometry using a BD LSRFortessa™ X-20 (BD Biosciences) or Aurora flow cytometer (Cytek Biosciences, Fremont, California, USA) and FlowJo™ 10 analysis software (BD Biosciences). Cells were enumerated using a standard amount of blank calibration particles (ProSciTech, Kirwan, Queensland, Australia) as determined using a hemocytometer [17].

For flow cytometric analysis of cell death and phenotype, BAL cells were treated with Fc receptor-blocking monoclonal antibody against CD16/CD32 (clone 93, Thermo Fisher Scientific) to limit non-specific antibody binding, followed by staining with fluorochrome-conjugated monoclonal antibodies as above. In some experiments, monoclonal antibodies to CD11b (clone M1/70, BioLegend) and CD69 (clone H1.2F3, BD Biosciences) were also included. Cells were then incubated with Annexin V (BioLegend) in binding buffer (10 mM HEPES pH 7.4, 150 mM NaCl, and 2.5 mM CaCl_2_) and 5 µg mL^−1^ propidium iodide (PI; Thermo Fisher Scientific). Cells were analyzed using a BD LSRFortessa™ X-20 (BD Biosciences) or Aurora flow cytometer (Cytek Biosciences, Fremont, California, USA) and FlowJo software (BD Biosciences).

### Immunofluorescence and immunohistochemical staining of lung tissue sections

In the indicated experiments, mice were sacrificed via intraperitoneal injection of sodium pentobarbital, and lungs were immediately inflated and fixed in 10% neutral buffered formalin (NBF) for at least 24 h, and then processed in paraffin wax. Longitudinal lung tissue sections (4 µm) were dewaxed and rehydrated, then microwaved for 6 mins in citrate buffer (10 mM citrate, pH 6) for heat-induced antigen retrieval. Lung sections were incubated in CAS-Block Histochemical Reagent (Thermo Fisher) for 1 hr and then incubated overnight with primary antibodies against cleaved Gsdmd (E3E3P, Cell Signaling Technology) and E-Cadherin (AF648SP, R&D Systems). Sections were then stained with anti-goat and anti-rabbit secondary antibodies (Thermo) for 1 h followed by Hoechst 33342 nuclear staining (Thermo Fisher). Sections were mounted using Fluorescence Mounting Medium (Agilent, Santa Clara, USA). Slides were examined under a Nikon A1R confocal microscope (Nikon, Japan). Confocal imaging was performed at 40× magnification and images were processed using ImageJ (National Institute of Health, USA). The % of Hoechst^+^ cells that were positive for cleaved GSDMD per field of view (FOV) was quantified using ImageJ software.

In the indicated experiments, Terminal deoxynucleotidyl transferase-mediated dUDP nick-end labeling (TUNEL) assay was performed on lung tissue sections using the ApopTag Peroxidase In Situ Apoptosis Detection Kit (Merck Millipore), according to the manufacturer’s instructions. Sections were counterstained with hematoxylin. Lung sections were viewed on an Olympus BX60 microscope (Olympus, Tokyo, Japan) and photographed at 10× magnification with an Olympus DP74 color camera using Olympus cellSens Dimension software. TUNEL staining intensity (% positive pixel intensity per FOV) was quantified using ImageJ software. Color deconvolution was performed, and a threshold was set on DAB intensity, with the same parameters applied to all sections. Five random fields per section were analyzed.

### Assessment of lung damage and pathology

Levels of protein in BAL fluid supernatant were determined using a PierceTM BCA Protein Assay Kit (Thermo Fisher Scientific, Waltham, USA). Levels of LDH in BAL fluids were determined using a CytoTox 96 Non-radioactive Cytotoxicity Assay (Promega, Madison, USA), according to the manufacturer’s instructions.

In the indicated experiments, mice were sacrificed via intraperitoneal injection of sodium pentobarbital, and lungs were immediately inflated and fixed in 10% NBF for at least 24 h, and then processed in paraffin wax. Longitudinal tissue sections (4 µm) were prepared and stained with hematoxylin and eosin (H&E). Tissues were graded for alveolitis and peribronchial inflammation on a subjective scale of 0 to 5 (0 = no inflammation, 1 = very mild, 2 = mild, 3 = moderate, 4 = marked, and 5 = severe inflammation) [17, 19]. Sections were also scored for features of epithelial damage such as presence of debris in the airspace, epithelial denudation, and thickening of the epithelial wall (0 = no obvious damage, 1 = mild, 2 = moderate, 3 = marked, and 4 = severe) [17]. Sections were blinded and randomized, and samples corresponding to the least severe and most severe were assigned scores of 0 and 4/5, respectively. All other samples were graded in 5 random fields by two independent readers. Lung sections were viewed on an Olympus BX60 microscope and photographed at 10x magnification with an Olympus DP74 color camera using Olympus cellSens Dimension software.

### Data and statistical analysis

Sample sizes were estimated based on previous extensive experience in the laboratory within similar studies. The investigator was blinded to the group allocation for the assessment of histology but was not blinded for other experiments. No samples were excluded from analysis. Data were tested for normality and analyzed by GraphPad Prism version 9 software (Graphstats Technologies, Bangalore, India). A Student’s *t* test (two-tailed unpaired) was used when comparing two values. When comparing three or more sets of values, a one-way analysis of variance (ANOVA) was used with either Tukey’s or Dunnett’s multiple comparisons post-hoc test. Survival proportions were compared using the Mantel-Cox log-rank test. A *P* value < 0.05 was considered statistically significant. No samples were excluded from analysis.

## Results

### IAV infection promotes GSDMD cleavage in lung epithelial cells

**Fig. 1:**
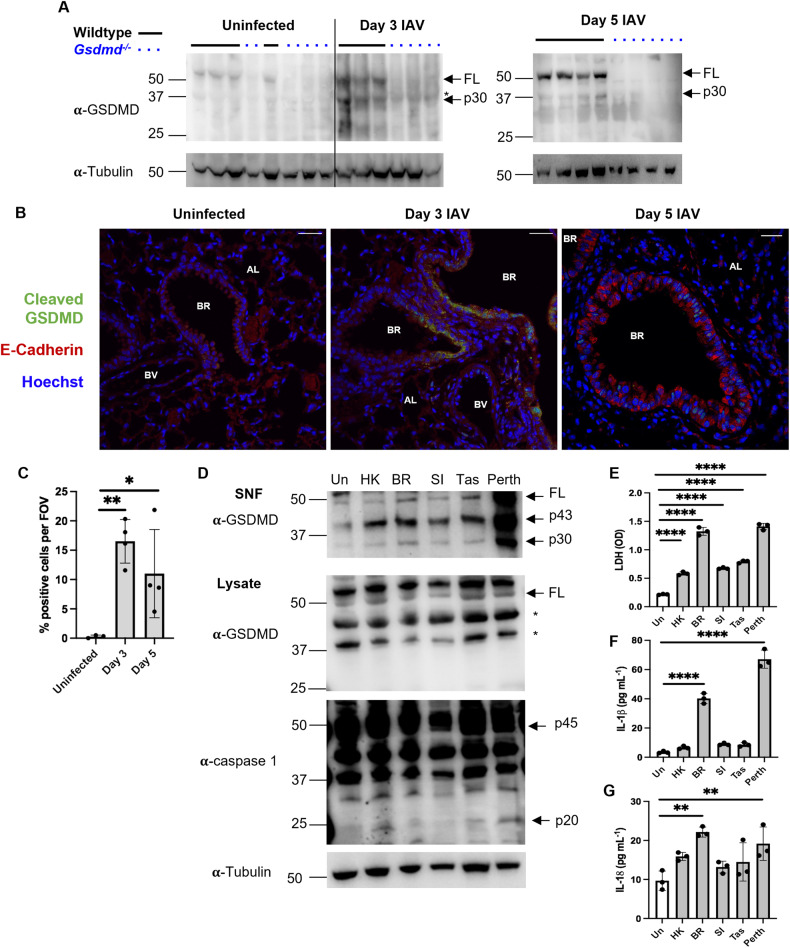
IAV infection results in cleavage of GSDMD in lung epithelial cells. **A**, **B** Wildtype and *Gsdmd*^−*/*−^ mice were infected with 10^4^ PFU of HKx31 IAV. **A** Immunoblot of GSDMD and tubulin protein in lung tissue lysates from uninfected and IAV-infected mice on day 3 and 5. Please note lanes 4 and 5 were inversely loaded unintentionally. Arrows indicate full length (FL) and active N-terminal p30 GSDMD subunit. Individual wildtype (horizontal solid black lines) and *Gsdmd*^−/−^ (dotted blue lines) mice are shown. * indicates non-specific band. Data are representative of one of two independent experiments, each consisting of 3 or 4 mice per group. **B** Expression of cleaved GSDMD (green) in E-Cadherin^+^ (red) epithelial cells in lung tissue sections measured by confocal microscopy on day 3 and 5 post-infection. Hoechst nuclear stain is shown in blue. Images were analyzed with ImageJ software. Bronchiole (BR), alveolus (AL) and a blood vessel (BV) are labeled. Representative images at 40× magnification (scale bar = 20 μm). **C** Quantification of cleaved GSDMD staining determined using ImageJ software. Data presented as the mean % positive cells per field of view (FOV) ± SD, with each data point representing an individual animal. *n* = 3 or 4 per group. *p < 0.05, **p < 0.01, one-way ANOVA. **D**–**G** Human normal bronchial epithelial HBEC3-KT cells were infected with human IAV: HKx31 (HK; H3N2), Brazil/78 (BR; H1N1), Solomon Islands/04 (SI; H1N1), Tasmania/09 (Tas; pandemic H1N1), and Perth/09 (Perth; H3N2). Data are representative of one of two independent experiments. **D** Immunoblot of GSDMD in cell supernatants (SNF; top) and cell lysates (bottom) at 24 h post-infection. Uninfected (Un) cells are shown. Arrows indicate full length (FL), and active p43 and p30 subunits of GSDMD. * indicates non-specific bands. Immunoblot of pro-caspase 1 (p45), caspase-1 subunit p20 and tubulin protein in cell lysates is also shown. **E** Levels of LDH and **F** IL-1β and **G** IL-18 in cell supernatants at 24 h post-infection, determined by colorimetric assay (OD; optical density) and ELISA, respectively. Data shown as experimental triplicates ± SD. **p < 0.01, ****p < 0.0001, One-way ANOVA.

GSDMD cleavage or activation is a hallmark feature of pyroptosis. To examine cleavage of GSDMD in vivo during IAV infection, we utilized a well-established preclinical model of severe IAV infection [7–9, 17]. Wildtype and *Gsdmd*^−/−^ mice were intranasally infected with 10^4^ PFU of HKx31 H3N2 IAV. *Gsdmd*^−/−^ mice [16] were kindly provided by Vishva Dixit (Genentech USA). Whole lung tissue lysates from IAV infected mice were evaluated for GSDMD expression and cleavage by immunoblot and compared against uninfected counterparts. IAV infection resulted in increased expression of full-length GSDMD on days 3 and 5 (Fig. 1A). Critically, the cleaved active N-terminal p30 subunit of GSDMD was detectable in lung tissues on day 3 and 5, suggesting IAV infection promotes GSDMD cleavage in the lung. Interestingly, full-length GSDMD was detectable in BAL cell lysates on day 3 (predominantly macrophages and neutrophils [9, 17]); however, the active N-terminal p30 subunit of GSDMD was not detected (Fig. S1), suggesting that during IAV infection GSDMD cleavage in airway leukocytes is limited in vivo. As lung epithelial cells represent the major site of IAV infection and replication [20], we next examined the expression of cleaved GSDMD in lung tissue sections by confocal imaging (Fig. 1B, C, S2). E-cadherin^+^ epithelial cells lining the bronchioles and alveoli expressed cleaved GSDMD on day 3 post-infection, suggesting GSDMD is activated in lung epithelial cells in vivo during IAV infection. The % of cells positive for cleaved GSDMD trended lower on day 5 (Fig. 1C). Importantly, expression of cleaved GSDMD was not observed in lung tissues from *Gsdmd*^−/−^ mice (Fig. S2). Lastly, in vitro infection of normal human bronchial epithelial (HBEC-3KT) cells with human H1N1 and H3N2 IAV resulted in GSDMD cleavage, with active p30 and inactive p43 subunits of GSDMD detected in cell supernatants at 24 h following infection (Fig. 1D). Caspase 1 is a well-described activator of GSDMD and consistent with this, the active p20 subunit of caspase 1 was detected in human epithelial cells following IAV infection (Fig. 1D). Lastly, IAV infection was associated with the release of LDH (Fig. 1E), IL-1β (Fig. 1F) and IL-18 (Fig. 1G) in cell supernatants. Together, these data demonstrate IAV infection promotes activation of GSDMD in murine and human lung epithelial cells.

### Absence of GSDMD limits the severity of IAV infection in vivo

**Fig. 2:**
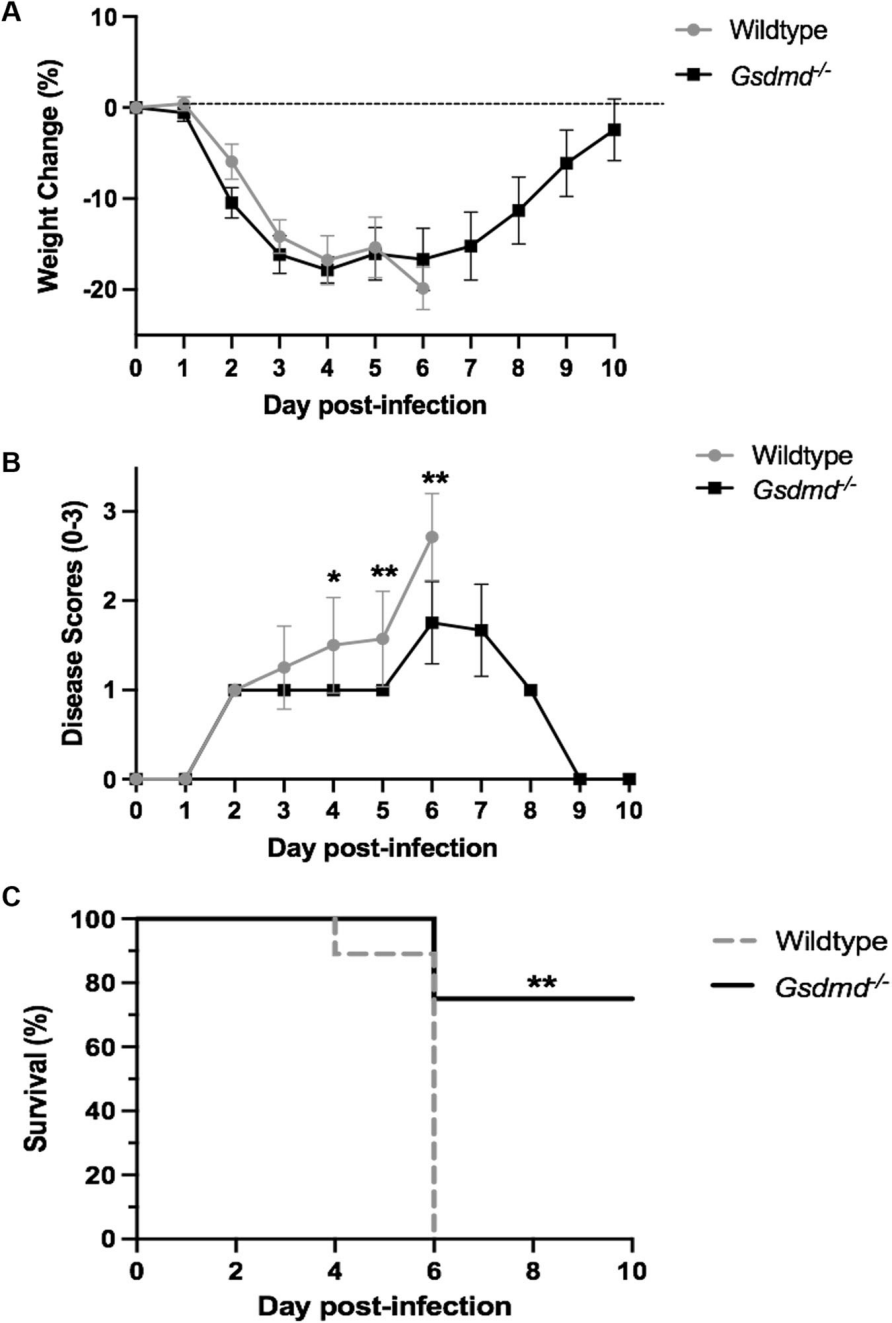
GSDMD deficiency limits the severity of IAV infection. Wildtype and *Gsdmd*^−/−^ mice were infected with 10^4^ PFU of HKx31 as a model of severe IAV infection. *n* = 8 per group, pooled from two independent experiments. **A** Mouse weights were recorded daily, and results are expressed as mean percent weight change ± SD. **B** Mice were scored daily for clinical signs of disease on a scale of 0–3, as described in the Materials and methods. Results are expressed as mean disease score ± SD. *p < 0.05, **p < 0.01, two-tailed unpaired Student’s *t* test. **C** Survival curves are shown. **p < 0.01, Mantel–Cox log-rank test.

Having established that IAV infection results in cleavage of GSDMD in lung epithelial cells (Fig. 1), we examined the susceptibility of *Gsdmd*^−/−^ mice to IAV infection. Wildtype and *Gsdmd*^−/−^ mice were infected with 10^4^ PFU of HKx31 (H3N2) IAV and monitored daily for weight loss and clinical signs of disease (score of 0-3, as described in the Materials and Methods). Mice were euthanized either upon losing greater than 20% of their initial body weight or displaying severe clinical signs of disease (score of 3). By day 6 post-infection, all wildtype mice had developed severe IAV disease characterized by 20% weight loss (Fig. 2A), reduced mobility, and rapid breathing (disease score of 3; Fig. 2B) and were subsequently euthanized (Fig. 2C). In contrast, *Gsdmd*^−/−^ mice developed less severe IAV disease as seen by reduced clinical disease scores (scores of 1–2; Fig. 2B). Critically, 75% of *Gsdmd*^−/−^ mice recovered from the infection by day 10 post-infection (Fig. 2C). These results suggest GSDMD deficiency promotes resistance to severe IAV infection.

### GSDMD deficiency limits neutrophil infiltration into the airways during IAV infection

**Fig. 3:**
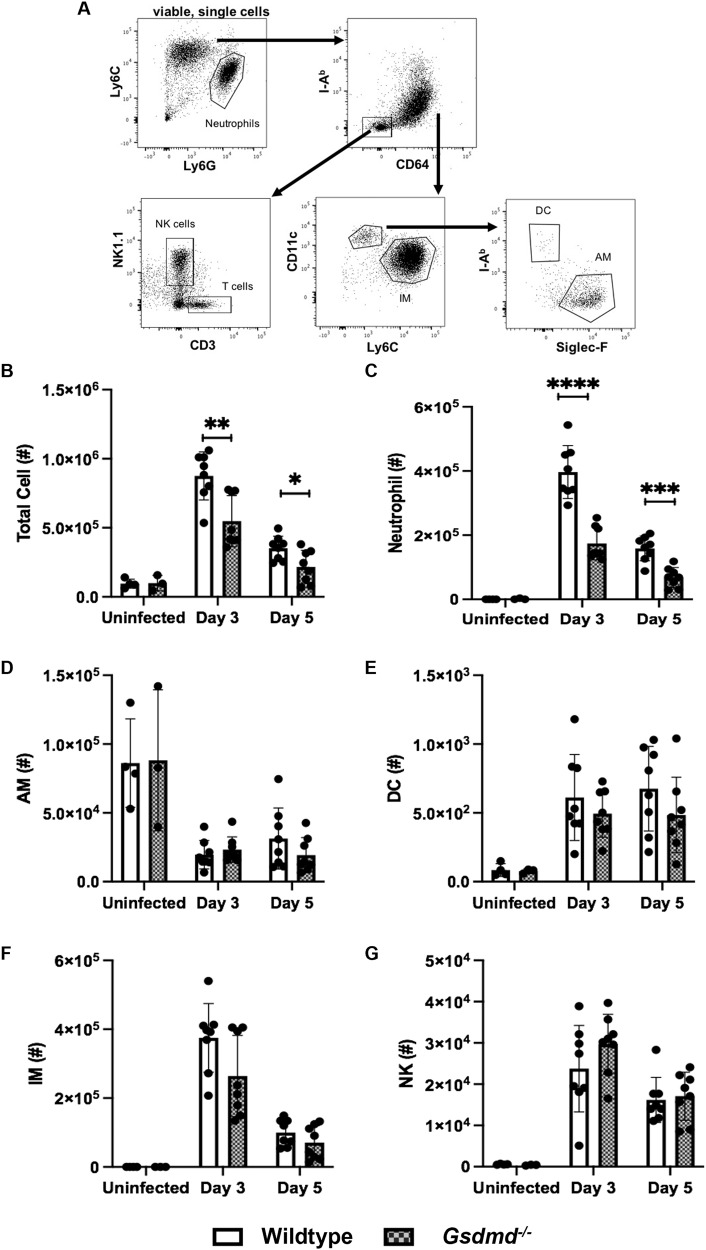
GSDMD deficiency limits the infiltration of neutrophils into the airways. Wildtype and *Gsdmd*^−/−^ mice (*n* = 8) were infected with 10^4^ PFU of HKx31 IAV and BAL performed on day 3 and 5 post-infection. Uninfected controls (*n* = 3 or 4) were included for comparison. **A** Representative flow cytometry gating strategy for BAL cells. **B** Numbers (#) of total live cells. Numbers (#) of live **C** neutrophils, **D** alveolar macrophages (AM), **E** dendritic cells (DC), **F** inflammatory macrophages (IM), and **G** natural killer (NK) cells, as determined by flow cytometry. Data are presented as the mean ± SD, pooled from two independent experiments, with each data point representing an individual animal. *p < 0.05, **p < 0.01, ***p < 0.001, ****p < 0.0001, two-tailed unpaired Student’s *t* test.

IAV infection induces the rapid infiltration of immune cells into the airways. The magnitude and nature of the cellular infiltrates may be critical factors contributing to morbidity and mortality during IAV infection. Having established that *Gsdmd*^−/−^ mice are more resistant to developing severe IAV disease (Fig. 2), we enumerated cellular infiltrates in BAL fluids at days 3 and 5 post-infection using flow cytometry (Fig. 3A). Total airway cellularity and numbers of neutrophils, natural killer (NK) cells, inflammatory macrophages (IM), dendritic cells (DC), and resident alveolar macrophages (AM) were determined [17]. Compared with wildtype mice, total BAL leukocyte numbers were significantly reduced on day 3 and 5 in infected *Gsdmd*^−/−^ mice (Fig. 3B). This correlated with a significant reduction in neutrophil numbers in the airways on day 3 and 5 post-infection (Fig. 3C). Resident AMs are susceptible to HKx31 IAV infection [20–22] and play an important protective role in vivo [21]. Consistent with our recent findings [17], IAV infection reduced AM numbers in the airways of wildtype mice on day 3 and 5 post-infection and a similar reduction was observed in the absence of GSDMD (Fig. 3D). Lastly, a trend for reduced IM numbers was seen on day 3 in the absence of GSDMD, with similar numbers of NK cells and DCs observed (Fig. 3E–G).

We next sought to correlate alterations in leukocyte numbers with possible changes in cell activation and/or cell death. A mild reduction in the surface expression of activation marker CD69 was seen on AMs in the airways of *Gsdmd*^−/−^ mice (Fig. S3A); however, CD69 expression on IMs was comparable between wildtype and *Gsdmd*^−/−^ cohorts (Fig. S3B). The reduced neutrophil numbers observed in *Gsdmd*^−/−^ mice (Fig. 3C) was not associated with changes in surface expression of activation markers CD69, CD11b, and MHC Class II on neutrophils (Fig. S3C–E). Interestingly, Annexin V and PI staining revealed GSDMD deficiency was not associated with a reduction in the frequency of dying (Annexin V^+^ PI^−^) or dead (Annexin V^+^ PI^+^) AMs, IMs, or neutrophils in the airways (Fig. S2F–H). Together, these results suggest GSDMD plays a major role in promoting the infiltration of neutrophils into the airways.

### GSDMD deficiency constrains excessive pro-inflammatory mediator production in the airways during IAV infection

**Fig. 4:**
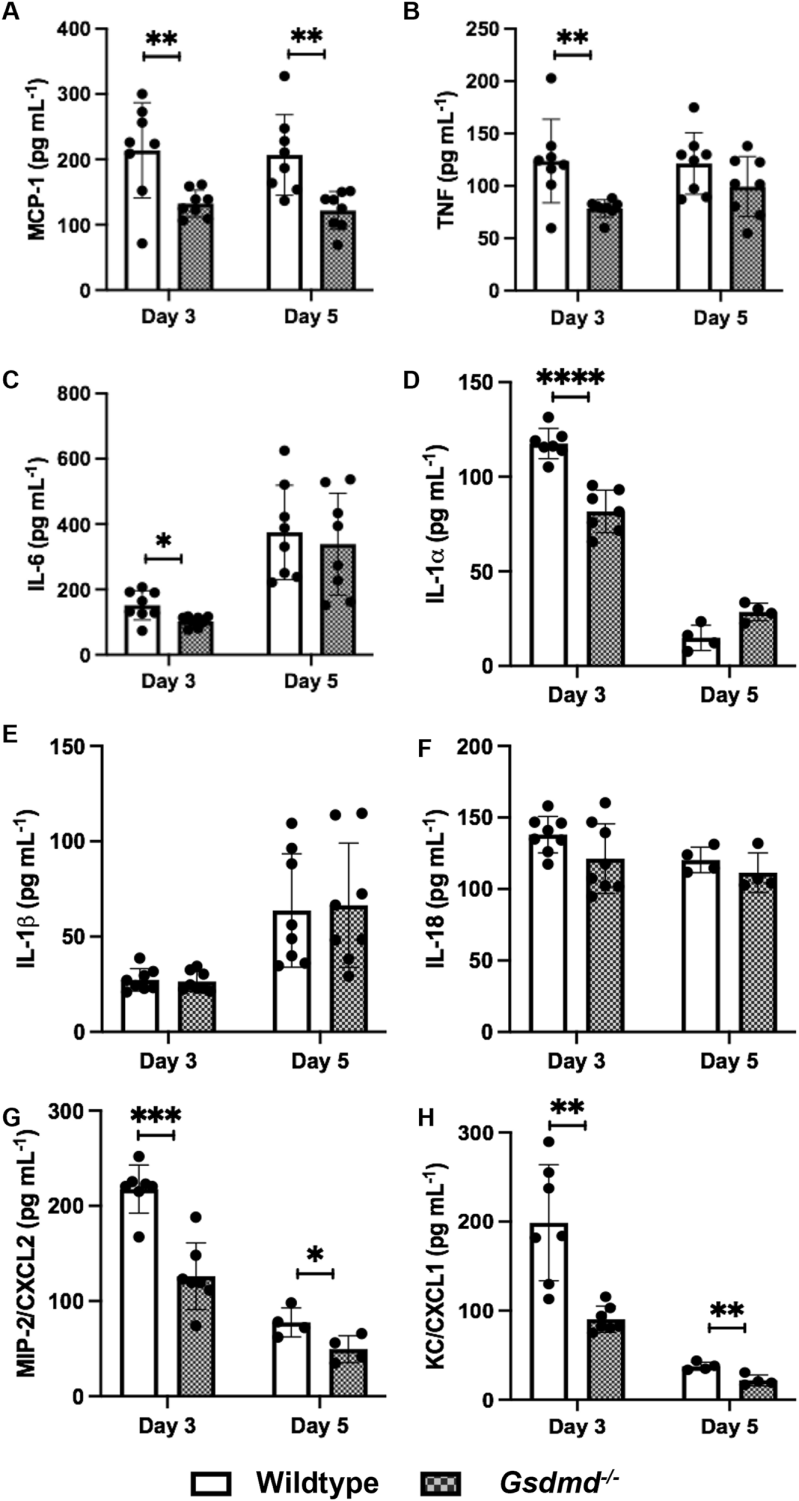
GSDMD deficiency limits the production of pro-inflammatory mediators in the airways. Wildtype and *Gsdmd*^−/−^ mice were infected with 10^4^ PFU of HKx31 IAV and BAL performed on day 3 and 5 post-infection. BAL fluid concentrations of **A** MCP-1, **B** TNF, **C** IL-6, **D** IL-1α, **E** IL-1β, **F** IL-18, **G** MIP-2/CXCL2, and **H** KC/CXCL1, determined by cytokine bead array or ELISA. Data presented as the mean ± SD, with each data point representing an individual animal (*n* = 4, 7, or 8 per group). Data from 1 to 2 independent experiments were pooled. *p < 0.05, **p < 0.01, ***p < 0.001, ****p < 0.0001, two-tailed unpaired Student’s *t* test.

Severe and fatal IAV infections are characterized by dysregulated and excessive cytokine responses, also known as ‘cytokine storm’. We next examined levels of pro-inflammatory mediators in the airways of wildtype and *Gsdmd*^−/−^ mice on days 3 and 5 following IAV infection. Levels of the pro-inflammatory cytokines IL-6, TNF, MCP-1, and IL-1α were all significantly reduced in *Gsdmd*^−/−^ mice on day 3 (Fig. 4A–D), with MCP-1 levels additionally reduced on day 5 (Fig. 4A). By contrast, no significant differences were observed in levels of interferon beta (IFNβ), interferon alpha (IFNα), interferon gamma (IFNγ), IL-10, or IL-12p70 at either time point (Fig. S4A–E). Interestingly, levels of NLRP3-dependent cytokines IL-1β, and IL-18 in the BAL were not altered by GSDMD deficiency (Fig. 4E, F). Immunoblot analysis of lung tissue lysates (Fig. S4F) and BAL fluids (Fig. S4G) confirmed no defect in IL-1β responses in *Gsdmd*^−/−^ mice, as expression of the mature IL-1β p17 subunit was comparable. Consistent with the observed reduction in neutrophil numbers in the BAL (Fig. 3C), neutrophil-attracting chemokines CXCL1 and CXCL2 were significantly reduced in BAL fluids from *Gsdmd*^−/−^ mice (Fig. 4G, H). Collectively, these data demonstrate GSDMD promotes excessive pro-inflammatory mediator production in the airways.

### Absence of GSDMD limits viral dissemination, epithelial damage, and pulmonary pathology during severe IAV infection

**Fig. 5:**
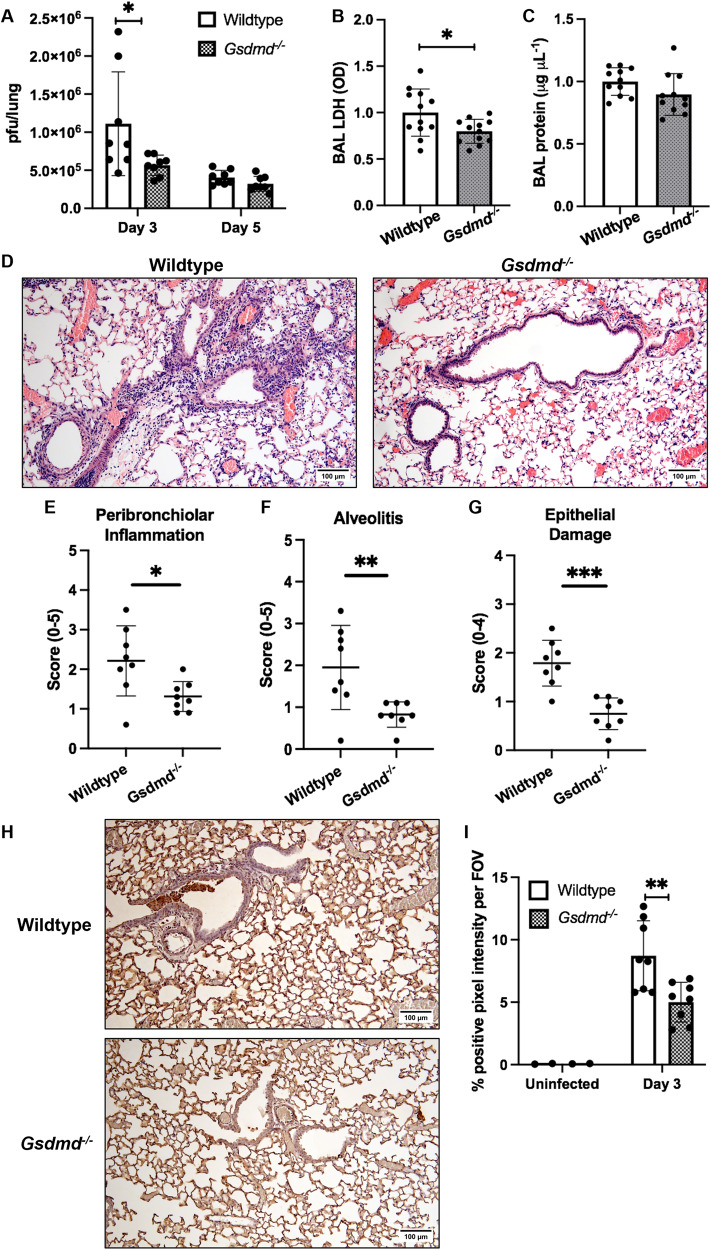
GSDMD deficiency limits viral loads, epithelial cell death, and immunopathology. Wildtype and *Gsdmd*^−/−^ mice were infected with 10^4^ PFU of HKx31 IAV. **A** Lung viral loads (pfu/lung) measured by a standard plaque assay. Data presented as the mean ± SD, with each data point representing an individual animal. *n* = 8 per group, pooled from two independent experiments. *p < 0.05, two-tailed unpaired Student’s *t* test. Rela*t*ive concentrations of **B** LDH (OD; optical density) and **C** total protein in BAL fluids at day 3, quantified using colorimetric assays. Data presented as the relative mean ± SD, with each data point representing an individual animal. *n* = 11 or 12 per group, pooled from three independent experiments. *p < 0.05, two-tailed unpaired Student’s *t* test. **D**–**G** Lungs were formalin inflated and fixed on day 3 post-infection. **D** Histological analysis of H&E-stained lung tissue sections were performed and representative images at 10× magnification (scale bar = 100 μm) are shown. Lung sections were randomized and scored blind for **E** peribronchial inflammation (scale 0–5), **F** alveolitis (scale 0–5) and **G** epithelial damage (scale 0–4), as described in the Materials and methods. Data are presented as the mean ± SD, with each data point representing an individual animal. *n* = 8 per group, pooled from two independent experiments. *p < 0.05, **p < 0.01, ***p < 0.001, two-tailed unpaired Student’s *t* test. **H**, **I** TUNEL assay labeling of cell death in lung tissue sections. **H** Representative images at 10× magnification (scale bar = 100 μm). *n* = 8 per group, pooled from two independent experiments. **I** Quantification of TUNEL staining determined using ImageJ software. Data presented as the mean % positive pixel intensity per field of view (FOV) ± SD, with each data point representing an individual animal. **p < 0.01, two-tailed unpaired Student’s *t* test.

Having established that GSDMD deficiency in vivo limits IAV-induced airway hyperinflammation (Fig. 3 and Fig. 4), we examined lung viral burden, tissue damage, and immunopathology. Lung infectious viral burden (pfu/lung) was significantly reduced at day 3 post-infection in mice lacking GSDMD, with a less profound reduction in viral loads observed at day 5 (Fig. 5A). Levels of LDH in BAL fluids were significantly lower in *Gsdmd*^−/−^ mice (Fig. 5B), with a trend for reduced total protein concentrations (Fig. 5C), collectively suggestive of diminished pulmonary damage in the absence of GSDMD. Histopathological analysis of H&E-stained lung tissue sections (Fig. 5D) indicated that peribronchial inflammation, alveolitis, and epithelial damage were significantly diminished on day 3 in *Gsdmd*^−/−^ mice (Fig. 5E–G). Lastly, TUNEL labeling revealed *Gsdmd*^−/−^ mice displayed significantly reduced tissue and epithelial cell death (Fig. 5H, I) [23]. Together, these data demonstrate GSDMD promotes pulmonary viral dissemination, tissue damage and cell death during severe IAV infection.

## Discussion

Over the past century, pandemic IAV have demonstrated the catastrophic impact the emergence of a novel respiratory virus can have on global human health and economic stability. Seasonal IAV continue to cause yearly epidemics with considerable morbidity and mortality. Additionally, outbreaks of ‘bird flu’ have caused more than 2400 sporadic infections in humans, with an associated mortality rate of 40% [24, 25]. Current IAV antiviral drugs have shown limited efficacy [26], and without new effective and safe host-directed therapies, we will continue to be vulnerable to developing severe IAV disease.

The severity of IAV disease may be dictated by the maintaining of a balance between rapid virus elimination and regulation of the immune response to limit immunopathology. Severe IAV infections in humans are characterized by excessive inflammatory responses, cell death, and damage to the epithelium, which leads to fatal acute respiratory distress syndrome (ARDS)-like disease [2, 21, 27, 28]. While numerous studies have defined a critical role for apoptosis in limiting the severity of IAV infection (reviewed in [3]), here we report that GSDMD promotes epithelial cell death and excessive pulmonary inflammation, including the production of pro-inflammatory cytokines and the infiltration of neutrophils into the airways, leading to increased pulmonary damage and pathology.

In the lung, resident AMs and epithelial cells lining the airways are the primary targets of IAV infection, with epithelial cells representing the major site of viral replication [20, 21, 29]. In general, cell death pathways, such as those involving apoptosis, are thought to interrupt the IAV replication cycle and limit virus amplification. Consistent with this, IAV infection of primary murine and human macrophages is described as ‘abortive’, in which viral proteins are expressed within the cell but infectious virus is not released [20, 21, 30]. Macrophages therefore provide a ‘dead end’ important for the control of IAV infection, with in vivo depletion increasing viral loads and exacerbating disease [21]. Of note, the PR8 H1N1 IAV strain was adapted to mouse lung by >300 sequential passages [31], and as such, it poorly infects murine macrophages [20, 21]. Conversely, the HKx31 H3N2 IAV used in our study and human seasonal H3N2 and H1N1 IAV are more efficient at infecting macrophages [18, 21, 30]. Here we observed IAV infection depleted AM numbers in the airways (Fig. 3D) and induced IM infiltration (Fig. 3F) in wildtype and *Gsdmd*^−/−^ mice. Interestingly, we failed to detect cleaved GSDMD in BAL cell lysates from IAV-infected wildtype mice (Fig. S1), and GSDMD deficiency had limited impact on AM or IM cell death (Fig. S3F, S3G), which correlated with a similar abundance of these cell types in infected airways (Fig. 3D, F). Together, these data suggest that in the absence of GSDMD, macrophage cell death in vivo during IAV infection is largely unaltered. Conversely, IAV infection induced cleavage of GSDMD in vivo in lung epithelial cells (Fig. 1B and C) and in vitro in normal human bronchial epithelial cells (Fig. 1D). However, a limitation is the human bronchial epithelial cells were not grown in an air liquid interface and therefore more closely emulate basal cells rather than epithelial cells of the bronchus. In support of our findings, GSDMD cleavage has been reported in human precancerous respiratory epithelial cells (PL16T cells) following IAV infection [32]. Critically, in our study, mice lacking GSDMD displayed reduced epithelial cell damage (Fig. 5G) and death (Fig. 5H, I); key determinants of IAV-induced ARDS-like disease [27].

IAV infection of epithelial cells and macrophages results in the rapid recruitment of leukocytes into the airways, predominantly IMs and neutrophils (Fig. 3). We observed no significant differences in numbers of IMs, NK cells, and DCs in the airways of wildtype and *Gsdmd*^−/−^ mice following IAV infection. Interestingly, the rapid infiltration of neutrophils into the airways was significantly impaired in the absence of GSDMD (Fig. 3C). Notably, this was not associated with major alterations in neutrophil activation (Fig. S3C–E) or cell death (Fig. S3H) but rather a reduction in levels of neutrophil-attracting chemokines CXCL1 and CXCL2 (Fig. 4G, H), which are produced by IAV-infected epithelial cells [20]. We have previously shown potent and sustained antibody-mediated depletion of neutrophils prior to and throughout IAV infection increased viral burden and promoted the development of severe IAV-induced disease [19, 33]. However, treatment with 2 low doses of neutrophil-depleting antibodies has been shown to improve IAV infection outcomes in mice without altering viral burden [34]. As such, in contrast to complete depletion of neutrophils in the airways, limiting their numbers may be beneficial through a reduction in immunopathology. Consistent with *Gsdmd*^−/−^ mice displaying improved resistance to severe IAV infection (Fig. 2), as well as the observed reduction rather than ablation of neutrophil infiltrates (Fig. 2C), limiting GSDMD activation during IAV infection may therefore fine-tune neutrophil responses in the airways.

Cytokines play a critical role in mediating protective inflammation; however, excessive responses can promote cell death and tissue damage. In this study, the absence of GSDMD limited the production of several key pro-inflammatory mediators in the airways (Fig. 4), which correlated with increased resistance to infection, namely improved survival (Fig. 2), as well as reduced lung viral burden and pathology (Fig. 5). Critically, GSDMD-mediated cell death leads to cell lysis and the release of cellular contents, including PAMPs and DAMPs [35, 36], which have the potential to further promote inflammation via activation of toll-like receptors (TLRs) and the secretion of NFκB-dependent cytokines. Consistent with this, TNF, IL-6, and MCP-1, cytokines commonly associated with ‘cytokine storms’, were reduced in the airways of *Gsdmd*^−/−^ mice (Fig. 4A-C). DAMPs released following GSDMD-mediated cell lysis are also well-described NLRP3 inflammasome activators, which could feed forward to exacerbate GSDMD-mediated cell death. Thus, during severe IAV infection, GSDMD activity may overamplify the local innate immune response, contributing to damaging immunopathology and compromising the health of the host.

The GSDMD pore has also been shown to facilitate the release of NLRP3-dependent cytokines IL-1β and IL-18 from macrophages and dendritic cells [13, 14]. Interestingly, in vivo IL-1β and IL-18 responses were not altered in the absence of GSDMD (Fig. 4E, F, Fig. S4F, G). This observation may reflect a potential differential role for GSDMD in myeloid and epithelial cells and/or a potential role for GSDMD-independent release of IL-1β and IL-18 in vivo during IAV infection. More recently, GSDMD has been shown to promote the release of IL-1ɑ [37], which reportedly also supports neutrophil infiltration and promotes pulmonary damage during IAV infection [38]. Congruent with these findings, we observed reduced levels of IL-1ɑ in the airways of infected *Gsdmd*^−/−^ mice (Fig. 4D). GSDMD deficiency may therefore rebalance innate immune responses to limit tissue damage without compromising critical protective host immunity.

Together, our findings define a detrimental role for GSDMD in promoting damaging inflammation and the development of severe IAV disease. Critically, IAV infection induced GSDMD cleavage in lung epithelial cells with mice lacking GSDMD displaying reduced inflammatory cell death, viral burden, and damage to the epithelium. There is an urgent need to develop new treatment for respiratory viruses, particularly those with pandemic potential. Novel host-directed therapies that limit hyperinflammation-associated morbidity and mortality, a common feature of severe respiratory virus infections, are an increasingly attractive option compared to current IAV antivirals, which can lead to the emergence of drug resistant IAVs. As such, GSDMD may be a new candidate therapeutic target for treatment of influenza and to improve disease outcomes.

### Supplementary information

Supplmental Figures

Orginal Data Files

Checklist

## Data Availability

All datasets generated and analyzed during this study are included in this published article and its Supplementary Information files. Full length uncropped western blots are available in the supplemental material files. Additional data are available from the corresponding author on reasonable request.
