# Supplementary material for: Gasdermin D promotes hyperinflammation and immunopathology during severe influenza A virus infection
Source: Cell Death Dis. 2023 Nov 9;14(11):727. doi: 10.1038/s41419-023-06258-1 (PMC10636052; doi:10.1038/s41419-023-06258-1)
Supplement: Supplementary file 1 — Supplmental Figures [file 41419_2023_6258_MOESM1_ESM.pdf]

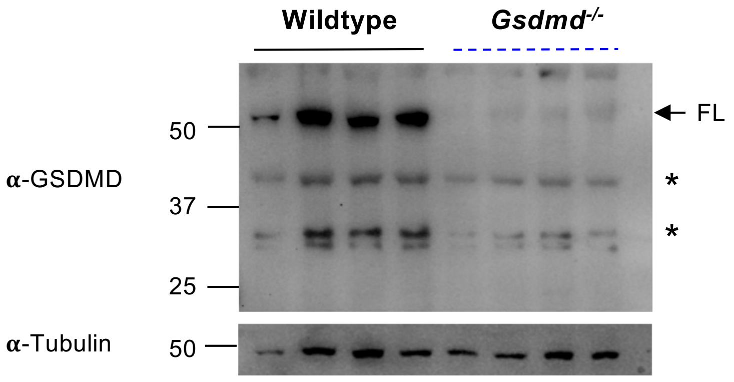

Fig. S1. GSDMD expression in BAL cells from the airways of IAV infected mice. Wildtype and *Gsdmd*$^{-/-}$ mice were infected with 10^4 PFU of HKx31 IAV. BAL cells were harvested on day 3 post-infection. Immunoblot of GSDMD and tubulin protein. Arrow indicates full-length (FL) GSDMD. The p30 GSDMD subunit was not resolved. * indicates non-specific bands. Data is representative of 2 independent experiments, each consisting of n=4 per group.

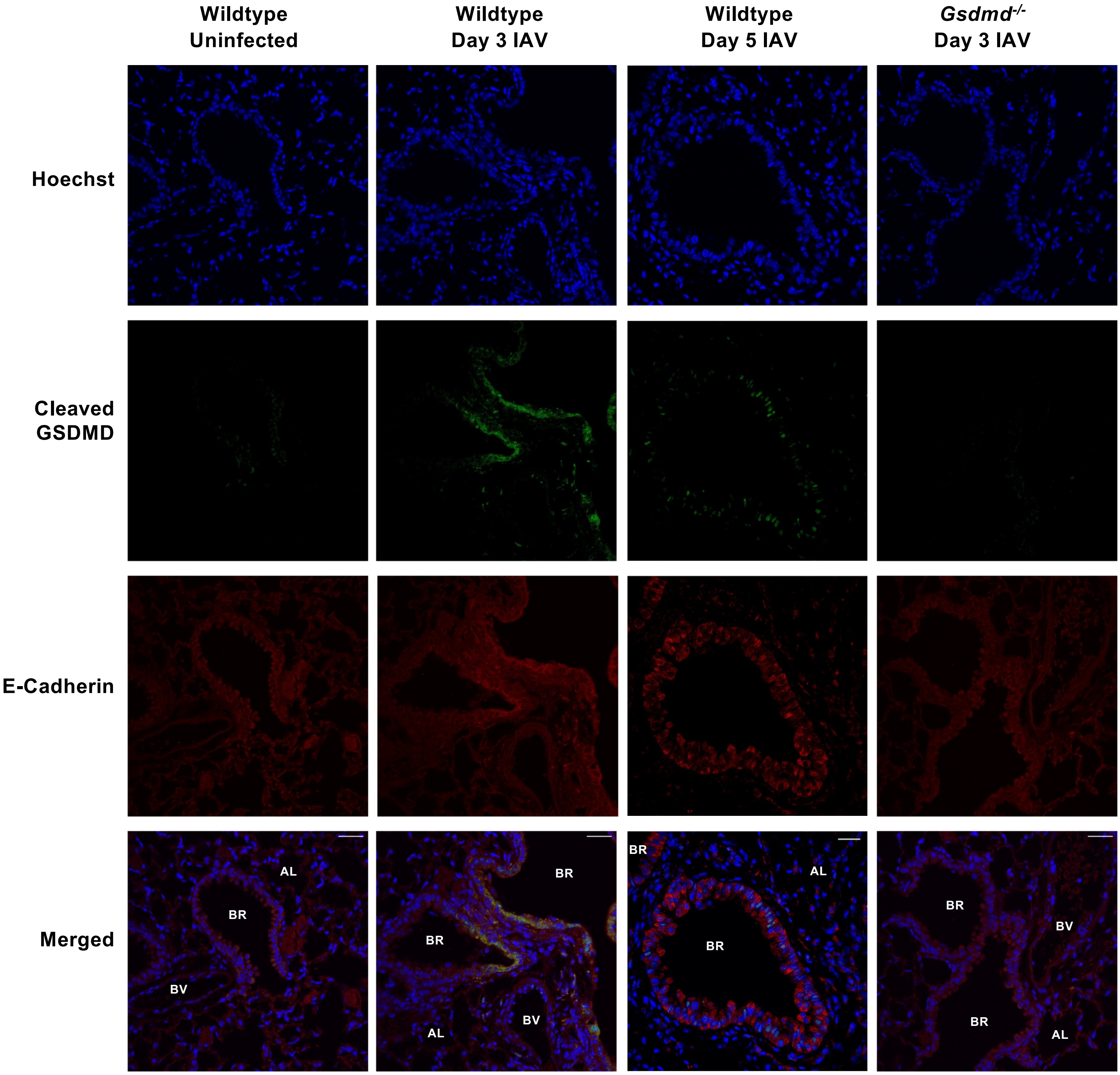

Fig. S2. Expression of cleaved GSDMD in lung epithelial cells following IAV infection. Wildtype and *Gsdmd*$^{-/-}$ mice were infected with 10^4 PFU of HKx31 IAV (n=3-4). Confocal imaging of lung tissue sections on day 3 and 5 post-infection. Expression of cleaved GSDMD (green) in E-Cadherin$^+$ (red) epithelial cells. Hoechst nuclear stain is shown in blue. Images were analyzed with ImageJ software. Bronchiole (BR), alveolus (AL) and a blood vessel (BV) are labeled. Scale bar = 20 µm.

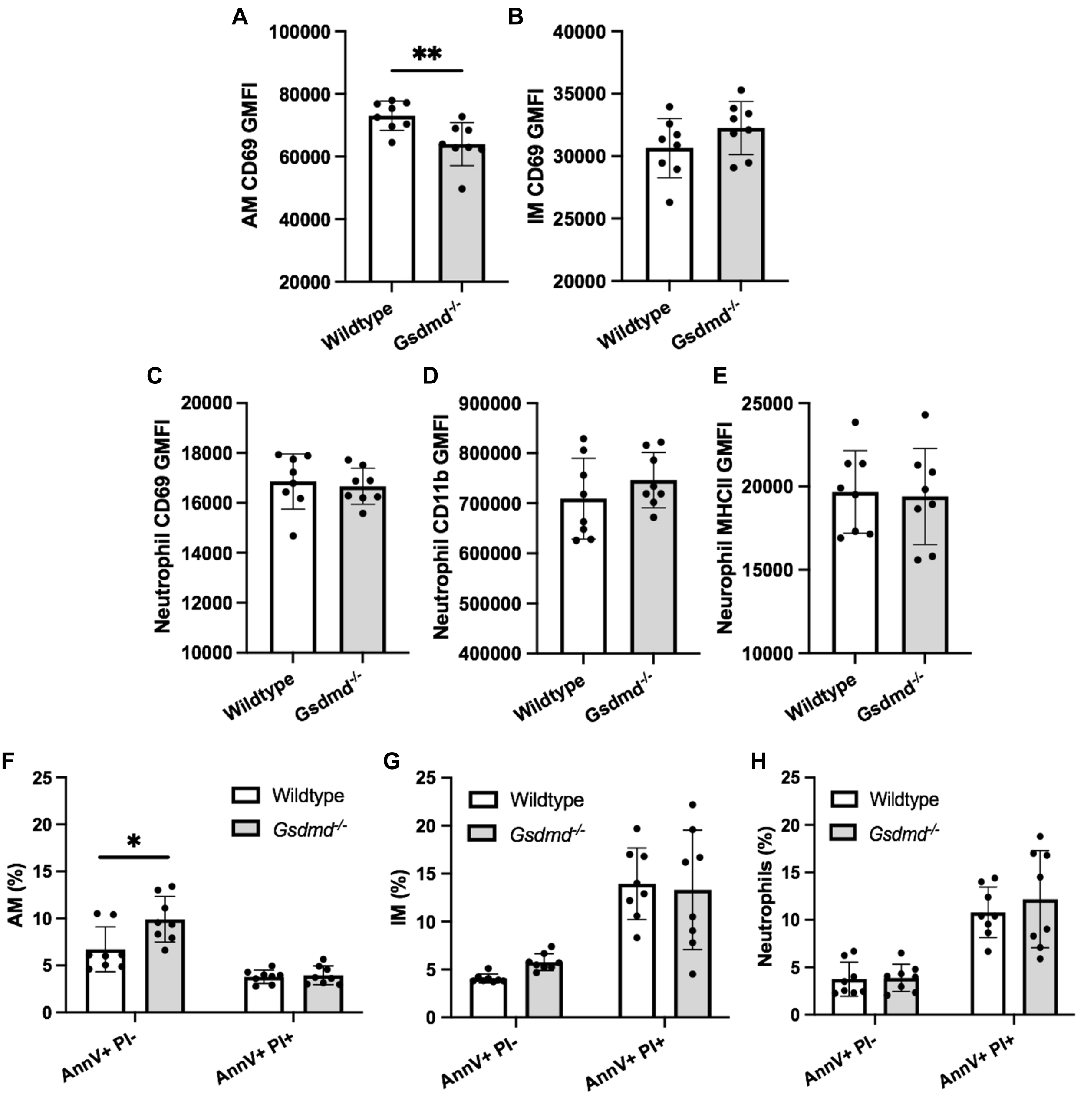

Fig. S3. Immune cell phenotype and cell death is not largely altered in the absence of GSDMD. Wildtype and *Gsdmd*$^{-/-}$ mice were infected with 10^4 PFU of HKx31 IAV and BAL cells analyzed by flow cytometry on day 3 post-infection. Expression of activation marker CD69 on (A) alveolar macrophages (AMs), (B) inflammatory macrophages (IMs) and (C) neutrophils. Expression of (D) CD11b and (E) MHC Class II (MHCII) on neutrophils. (A-D) Expression presented as geometric mean fluorescence intensity (GMFI). Percentage of (F) AM, (G) IM, and (H) neutrophils that were Annexin V^+ PI^- ($AnnV^+$ PI^-) and Annexin V^+ PI^+ ($AnnV^+$ PI^+) in the BAL. (A-H) Data presented as the mean ± SD, with each data point representing an individual animal (n=8 per group). *p < 0.05, *Student's t-test.

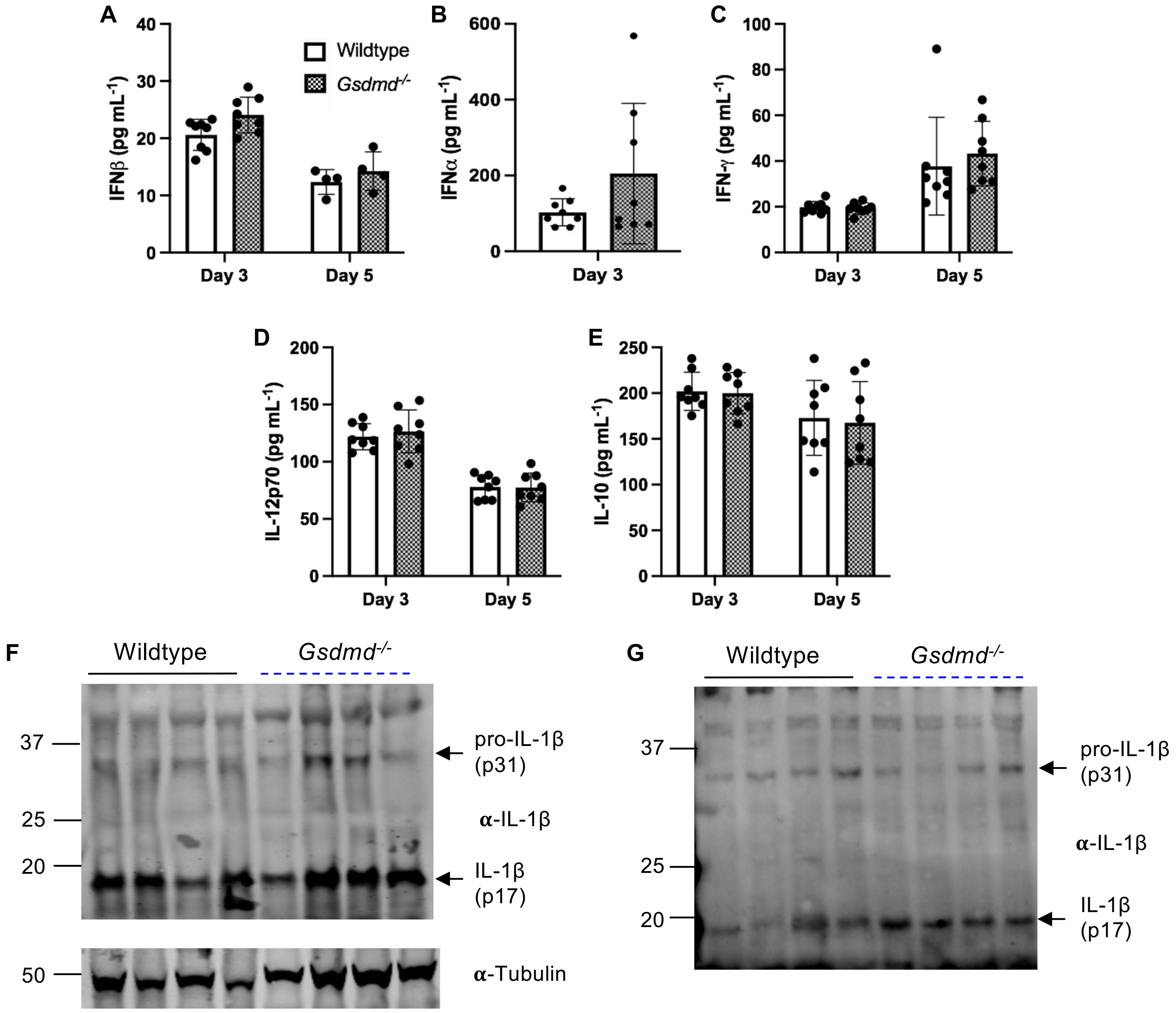

Fig. S4. Comparable levels of IFNβ, IFNγ, IL-10, IL-12, and IL-1β in the absence of GSDMD. Wildtype and *Gsdmd*$^{-/-}$ mice were infected with 10^4 PFU of HKx31 IAV. Levels of (A) IFNβ, (B) IFNα, (C) IFNγ, (D) IL-12p70 and (E) IL-10 in BAL fluids on day 3 and 5 post-infection. Data are presented as the mean ± SD, pooled from 1-2 independent experiments, with each data point representing an individual animal (n=4-8). Immunoblot of (F) lung tissue and (G) concentrated BAL fluid for pro-IL-1β (p31), IL-1β (p17), and tubulin protein. Data is representative of 2 independent experiments, each consisting of n=4 mice per group.
