# Supplementary material for: Gasdermin D promotes hyperinflammation and immunopathology during severe influenza A virus infection
Source: Cell Death Dis. 2023 Nov 9;14(11):727. doi: 10.1038/s41419-023-06258-1 (PMC10636052; doi:10.1038/s41419-023-06258-1)

Full Blots of Fig. 1A

α-GSDMD

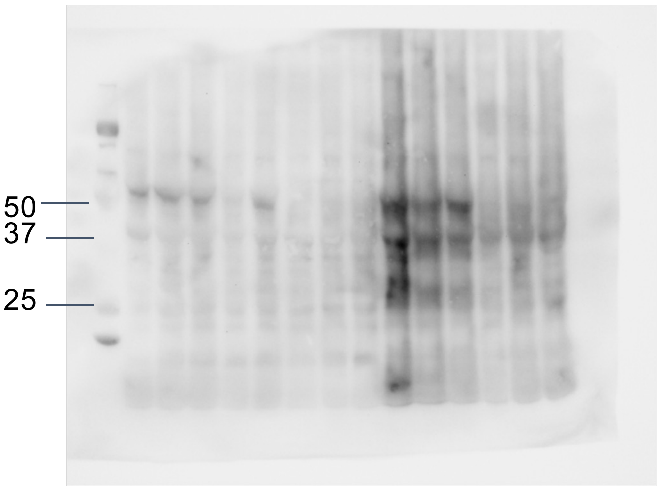

α-Tubulin

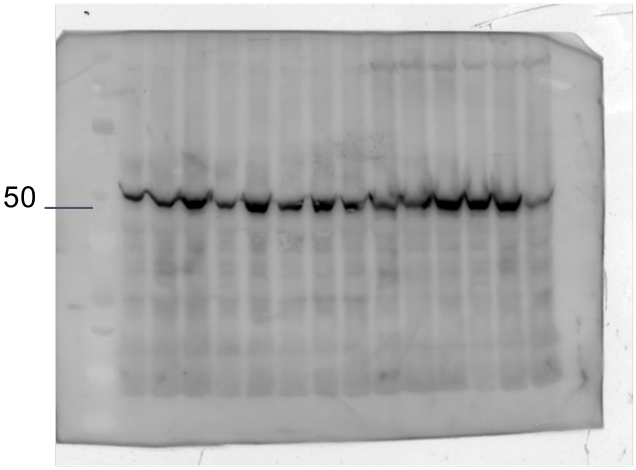

α-GSDMD

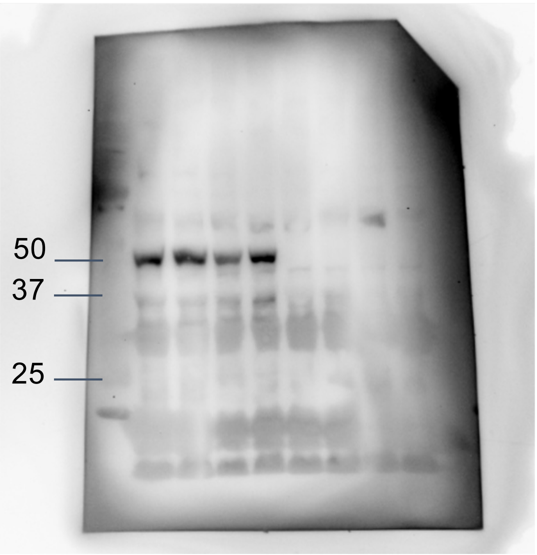

α-Tubulin

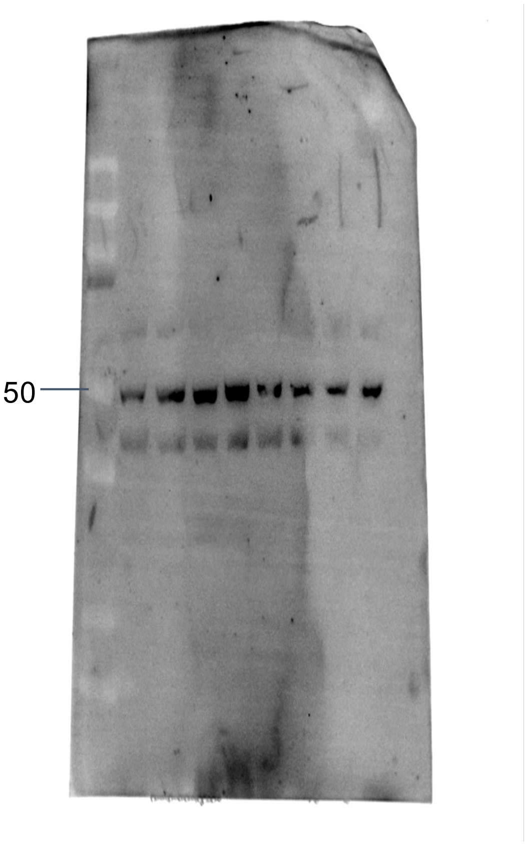

Full Blots of Fig. 1D

Lysate
α-GSDMD

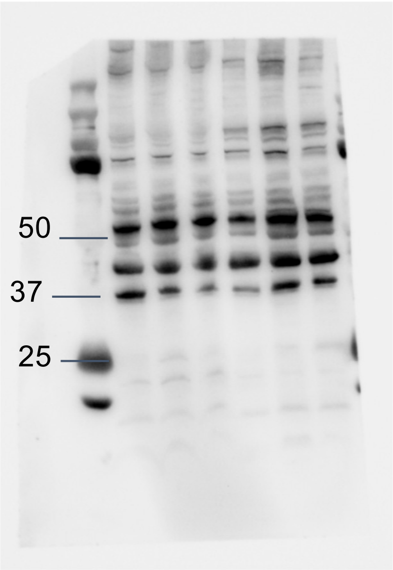

SNF
α-GSDMD

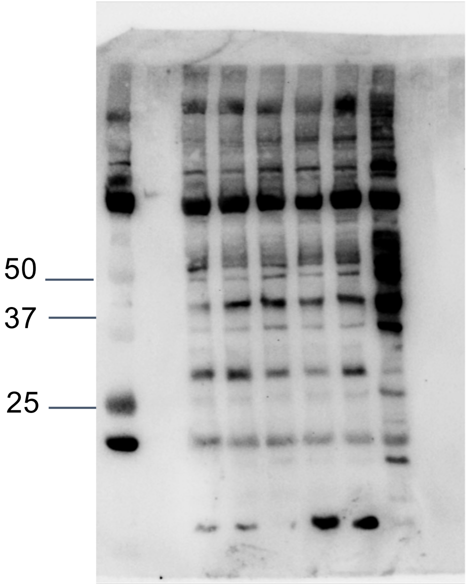

α-caspase 1

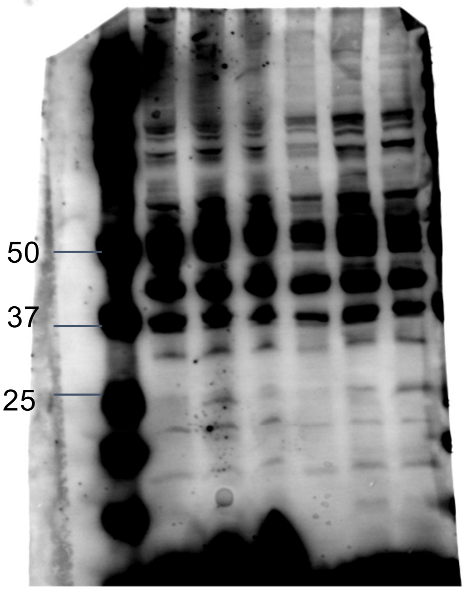

α-Tubulin

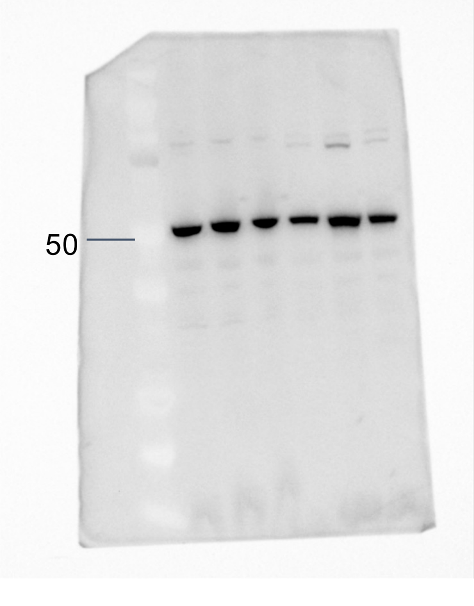

Full Blots of Fig. S1

α- GSDMD

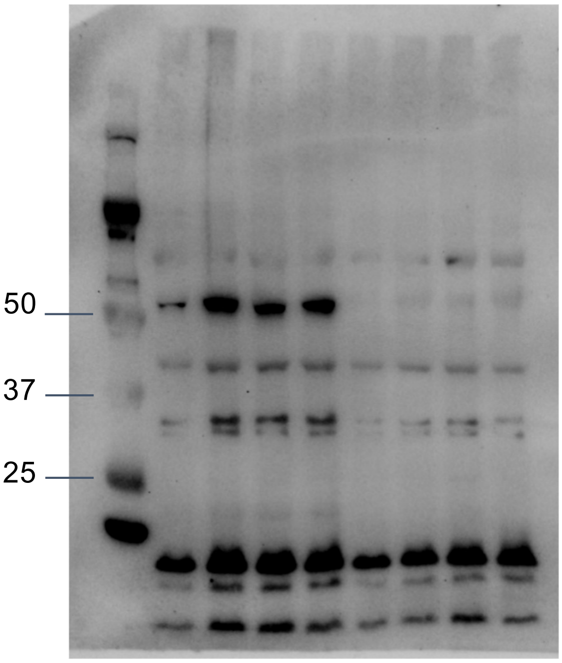

α-Tubulin

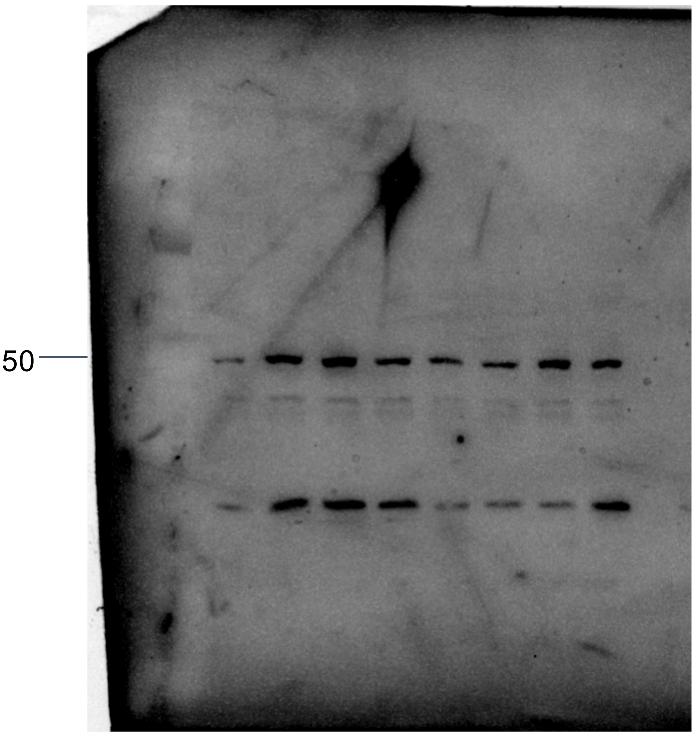

Full Blots of Fig. S4

α-IL-1β

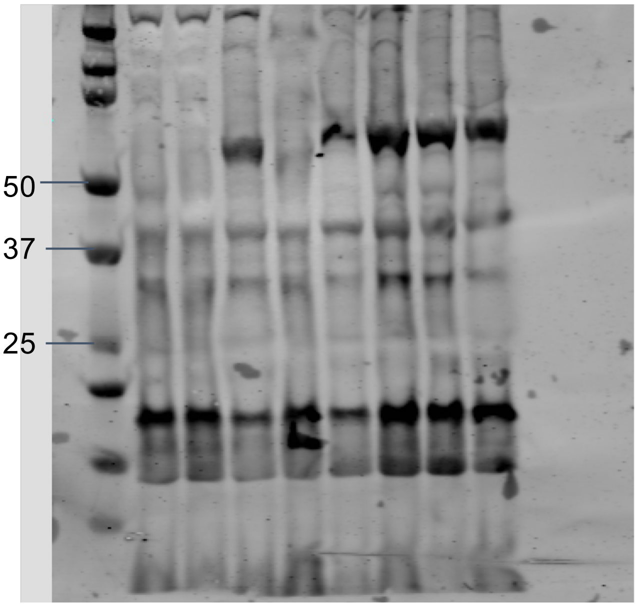

α-IL-1β

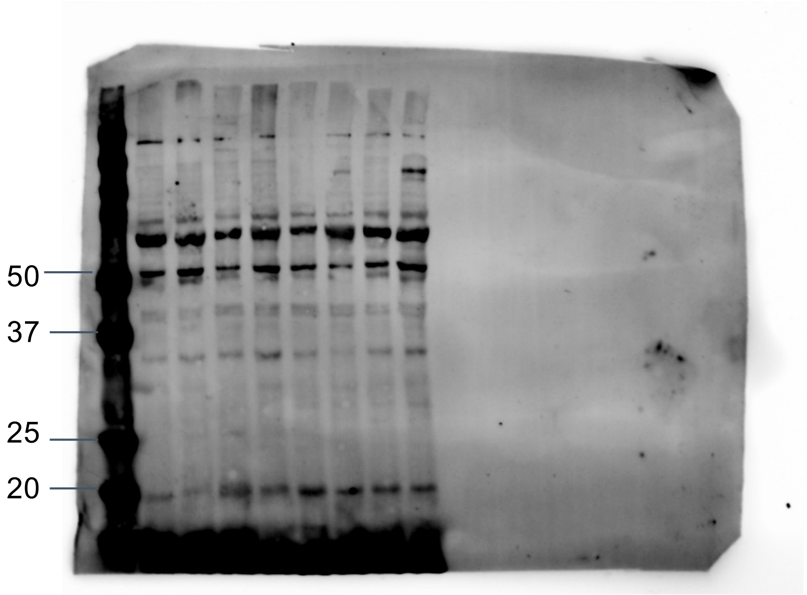

α-Tubulin

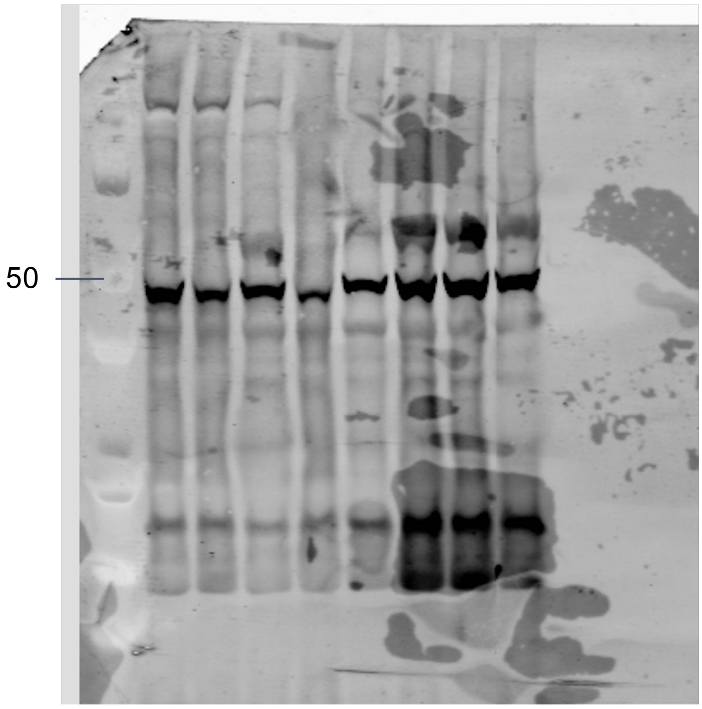

Supplement: Supplementary file 2 — Orginal Data Files [file 41419_2023_6258_MOESM2_ESM.pdf]
